# Clinical data and hearing of individuals with Alport syndrome

**DOI:** 10.1016/S1808-8694(15)30140-3

**Published:** 2015-10-18

**Authors:** Fatima Regina Abreu Alves, Fernando de Andrade Quintanilha Ribeiro

**Affiliations:** a PhD student in Otorhinolaryngology - Master in ENT - FCMSC-SP. Preceptor - ENT Ward - HSPM - SP; b PhD in Otorhinolaryngology - UNIFESP - EPM. Head Physician - Santa Casa de Misericórdia de São Paulo; Study developed at the Department of Otorhinolaryngology - Santa Casa de São Paulo

**Keywords:** hereditary, genetics, nephritis, hearing loss

## Abstract

Alport Syndrome (AS) is a hereditary disease, characterized by nephropathy, often times with sensorineural hearing loss and ocular defects. **Aim**: to analyze the clinical and hearing information from individuals with AS, more specifically the correlation between renal disorder and hearing loss (HL). **Study design**: clinical prospective with cross-sectional cohort. **Materials and Methods**: 37 individuals underwent otorhinolaryngological evaluation and were submitted to audiologic tests. For HL statistical analysis we considered only the results from the pure tone audiometries. **Results**: of the 28 individuals with clinical alterations, we found 46.4% of DLX and 53.6% of AD. HL happened to 46.1% of the individuals evaluated. 12 patients presented HL in the audiometric test: 11.5% mild and 34.6% moderate. Comparing the normal relatives with those with renal disorder; all that had HL also had renal disorder. In 30.8% the curve shape was mild descending in the high frequencies and in 11.5% it was flat. **Conclusions**: The inheritance pattern distribution does not match literature descriptions. HL is a frequent extra-renal finding. There is an association between renal involvement and HL (p= 0.009). The most frequent curve shapes: mild descending in the high frequencies and flat. There was no association between HL and age. There is no correlation between the HL and gender in this group.

## INTRODUCTION

**Figure 1:**
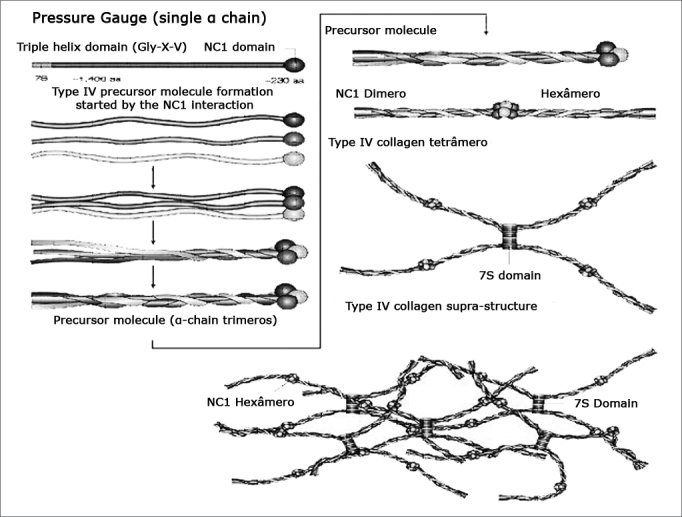
Type IV collagen structure. - Chains x3, x4 and x5(IV) are expressed on the basal membranes of kidneys, cochlea and eye. The mutations present in AS cause defects in these chains. The damage done to type IV collagen, caused by mutation, breaks its epithelial linkage function and causes organ damage. Modified from Kalluri, 2003. Gly=glycin; X=proline; Y=hydroxyproline; NC1= non-collagenous carboxy-terminal globular portion; 7S= amino-terminal domain; aa=amino acids.

Alport Syndrome (AS) is a hereditary disorder characterized by hematuria, which usually leads to renal failure. It can be followed by extra-renal manifestations. Therefore, often times, kidney disease is associated with sensorineural hearing loss (SNHL) and ocular effects.^1^ The dominant type is X related (DLX), happening in 85% of the cases, the autosomal recessive type (AR) happens in 10 to 15% of the cases, and the dominant autosomal (DA) type is rare.^2^ Improvements in molecular diagnosis may show that the DA type is not so uncommon.^3^ Mutations in the COL4A5 gene greatly, or possibly thoroughly, explain the X-related AS.4 In order to understand AS, it is necessary to understand the type IV collagen structure (Figure 1). 5 Type IV collagen is the main component of basal membranes. a3, a4 and a5 (IV) chains selectively express the basal membranes of some tissues, such as the kidney (glomerular basal membranes), the cochlea and the eye. Mutations present in AS cause defects on the a3, a4 and a5 (IV) chains. Such defects result in an intertwining and incorrect assemblage of monomers, which will be promptly degraded. The following signs are observed in the clinical settings of AS. Hematuria is a cardinal sign and it is seen in the first year of life. Proteinuria reflects a specific glomerular involvement, and it is absent in the first years of life. Renal failure ensues progressively. SNHL is of variable intensity, bilateral, symmetrical and progressive. The DLX type happens more frequently to men when compared to women. Ocular alterations: anterior lens cone (happens in 10 to 30% of the cases and it is pathognomonic of AS), ocular spots, cataract, nystagmus and myopia. Hematuria happens early on, SNHL evolves during the school years, and the final stage of renal failure and ocular alterations happens at the end of adolescence.^6^

## OBJECTIVES

The goal is to analyze clinical and hearing data from individuals with Alport Syndrome, with emphasis in the correlation between renal alteration and hearing loss.

## MATERIALS AND METHODS

This study and the Free Consent Form were approved by the Institution”s Ethics in Research Committee (Project # 118/05). This is a cross-sectional cohort clinical prospective study.

We assessed eight families and drew the respective genetic map, with information of at least one more generation. Clinical and audiologic data of the index cases (proband: members from which a family with genetic disorders is initially evaluated) referred were collected, and the family members who agreed to participate were evaluated. All the families matched at least two strict diagnostic criteria.

Thirty-seven individuals from these families agreed to take part in the study and carried out an assessment made of: detailed clinical and family history, complete otorhinolaryngological physical exam, assessing prior lab exam results (blood tests, urine, ultrasound and renal biopsy). Families 2, 5, 6 and 8 had individuals who underwent renal biopsy with histological alterations matching those found in individuals with Alport Syndrome. Patients did not have prior history of noise exposure nor of using ototoxic agents. Families 1, 4 and 5 had individuals with ocular alterations. We did not see any patient with anterior lens cone.

Audiological tests were carried out following the same standardization in index cases and in other family members who agreed to take part in the project. Children below three years of age who did not respond properly to the tests or could not be conditioned, for statistical purposes, were considered as not having gone through audiometric tests.

Results were classified, considering the mean value at 500, 1.000, 2.000 and 4.000 Hz, in the worst ear in: mild (21 - 40 dB HL); moderate (41 - 60 dB HL); moderately severe (61 - 80 dB HL); severe (81 - 100 dB HL); profound (> 100 dB HL) and not tested.^7^

A 15 dB difference between the ears in at least two frequencies or a 10 dB difference in four frequencies defines an asymmetrical hearing loss, and the symmetrical hearing loss has the same configuration and difference < 15 dB in the same frequencies between the ears.^7^

Audiometric curves were classified according to what was described on Table 1, in flat, sudden drop in the high frequencies, mild drop in the high frequencies, specific (U type or ascending) and residual.^8^

**Table 1:** Audiometric curve configuration classification.

Curve configuration	Definition
Flat	High, middle and low frequencies differ in less than 10 dBHL
Sudden drop in the high frequencies	The mean difference at 4.000, 6.000 and 8.000 and the mean value at 500, 1.000 and 2.000 Hz is higher than 25 dBHL, or the difference between any octave of a frequency is higher than 25 dBHL
Mild drop in the high Frequencies	The mean difference at 4.000, 6.000 and 8.000 and the mean value at 500, 1.000 and 2.000 Hz is between 10 and 24 dBHL
Residual	Remaining in the low frequencies
U type	Mid-range frequencies are worse than their high and low counterparts in 15 dBHL or more
Ascending	Low frequencies are worse than high frequencies in 10 dBHL or more

The genetic maps of the eight families were crafted from the data collected and with the results from the audiologic tests, separating the ones who took part in the study (in red) from those who only provide information.

Using the EpiInfo software, version 3.2, the following statistical tests were applied: chi-squared (c2) and Fisher Exact Test. We considered a 95% confidence interval in our statistical comparisons.

## RESULTS

1 - Genetic maps

Using standard symbols, the information obtained about the family history of the probands evaluated were graphically shown in the following genetic maps (Figure 2, Figure 3, Figure 4, Figure 5, Figure 6, Figure 7, Figure 8, Figure 9).

2 - Statistical analysis of the clinical and hearing data

**Figure 2:**
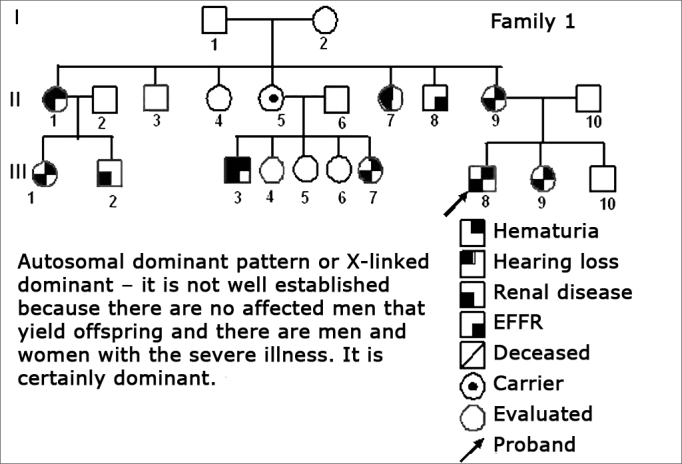
Family 1 Inheritance pattern - Probably autosomal dominant, with variable expression. Men and women are equally affected.

**Figure 3:**
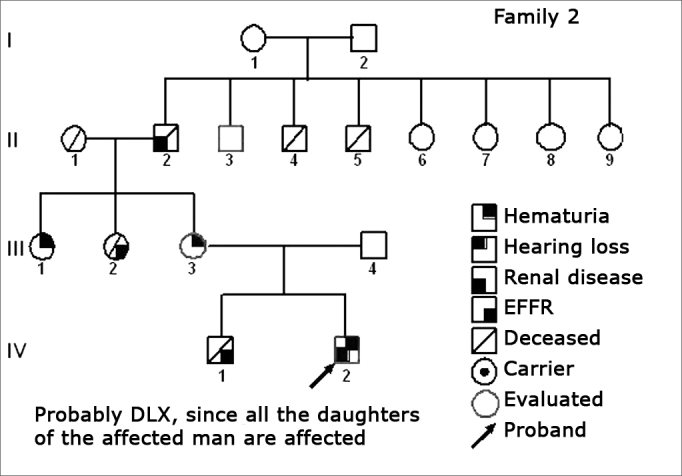
Family 2 Inheritance pattern - Probably DLX, because all daughters of the affected II-2 were affected.

**Figure 4:**
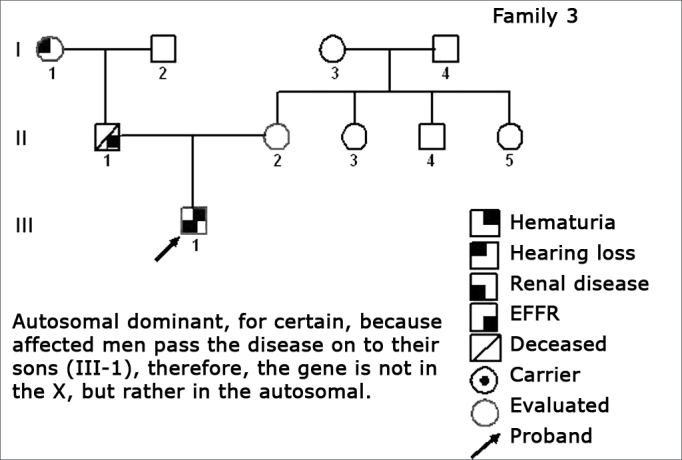
Family 3 Inheritance pattern - Autosomal dominant, because the affected man (II-1) passes on the disease for his son (III-1), therefore the gene is not in the X, but rather in a chromosome.

**Figure 5:**
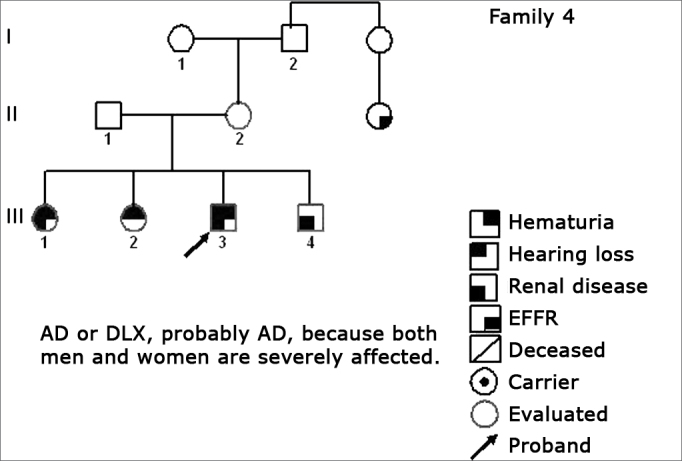
Family 4 Inheritance pattern - Probably AD. Cases III-1, III-3 and III-4 are very much diseased. DLX women have milder involvement than men.

**Figure 6:**
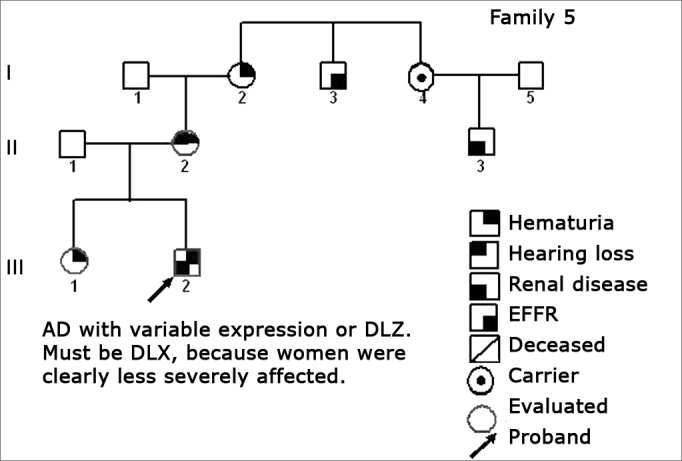
Family 5 Inheritance pattern - Probably DLX. The severity contrast between men and women matches this inheritance pattern.

**Figure 7:**
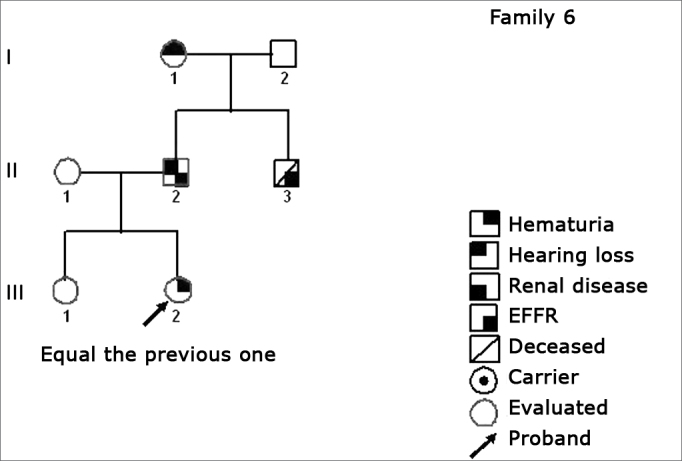
Family 6 Inheritance pattern - Probably DLX. The characteristic of a DLX inheritance pattern DLX, totally penetrating is that all the daughters and none of the men involved are affected.

**Figure 8:**
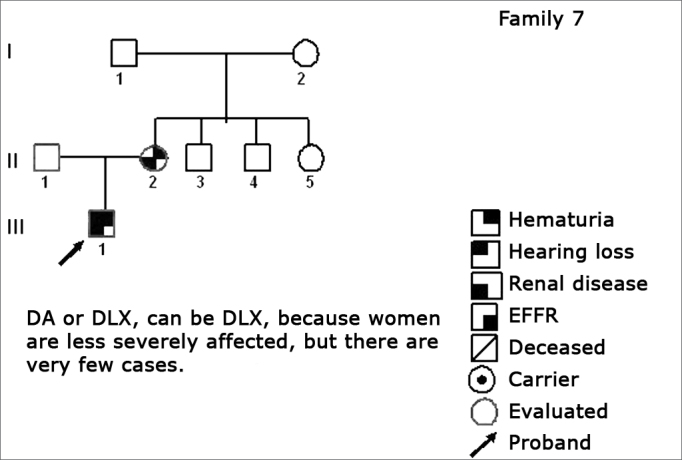
Family 7 Inheritance pattern - Probably DLX. Women are less affected; however there are fewer cases.

**Figure 9:**
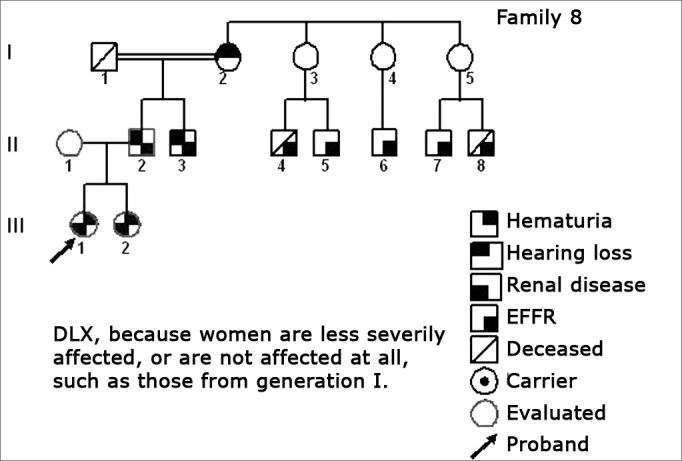
Family 8 Inheritance pattern - DLX, because women are less affected. All the daughters are less severely affect. All the daughters (III-1 and III-2) of the affected men (II-2) are affected, DLX characteristic.

Thirty-seven members from the eight families went through the evaluation approved by the Ethics in Research Committee of the Institution (Project # 118/05). The nine normal individuals were taken off the study.

Females prevailed in this group (16/28; 57.1%), and males were less commonly found (12/28; 42.9%).

Adults prevailed in this group of patients (12/28; 42.9%); pre-school and school aged children and teenagers together represent 50.0% (14/28).

Inheritance patterns were obtained after we created and analyzed the inheritance patterns. In this sample we found 46.4% of DLX and 53.6% of DA.

Twelve patients had hearing alterations in their audiometric tests: 11.5% (3/26) with mild alteration and 34.6% (9/26) with moderate alteration. Infants (below two years of age) who could not undergo the pure tone audiometry were taken off the analysis. In relation to the patients with mild hearing alterations age range (3/26): one was of school age (11 years) and two were adults. Of the patients with moderate alteration (9/26) the age distribution is the following: one school aged child (eight years), two teenagers (14 and 17 years) and six adults. Hearing loss happened in 46.1% (12/26) of the individuals evaluated. Five patients were referred to hearing aid fitting during our study.

We analyzed family members without clinical problems and we compared them with those that had renal disorder. All of those who had hearing problems also had renal involvement. There is an association between renal involvement and hearing impairment (p= 0.009). RD%: 20.36 -61.45. 52% (13/25) of the patients in any stage of the renal disease did not have alterations in their audiologic tests. 48% (12/25) of the patients at any stage of the renal disease manifested mild or moderate hearing disorder. From this analysis we excluded those infants (2/37) who did not undergo pure tone audiometry and one individual (1/37) who had not undergone urine tests during the study.

13 patients had normal audiometric curves (50.0%) and 30.8% have a mildly descending curve in the high frequencies (8/26). A flat curve happened in 11.5% (3/26). The less frequent configurations were: sudden drop in high frequencies and U type. Two infants were taken off the study.

In the group studied, although we observed 30.8% (8/26) with symmetrical hearing loss when both ears are compared, 19.2% (5/26) had asymmetrical hearing loss. Two infants were taken off the study.

We observed hearing alterations in the school-aged children, in the adolescents and in adults. We did not detect hearing changes in pre-school aged children. We took off the study those two infants who did not undergo pure tone audiometry. There was no statistically significant correlation between the hearing alteration and age among the patients studied.

In this group we observed 60.0% (6/10) of men with hearing disorder and 37.5% (6/16) of women with hearing disorder. There were 10/16 (62.5%) women without hearing disorder and 4/10 (40.0%) of men with the same problem. Two did not undergo audiometry because their ages were not suitable for the test. Literature reports that hearing loss happens, more frequently, in men when compared to women.

## DISCUSSION

In this study we had more women (57.1%) being evaluated than men (42.9%). In family 2 we noticed four males who died and in family 8 there were three members who died. This could also contribute to a lower participation of men in the study (Table 2).

**Table 2:** Individuals broken down by gender.

Gender	Frequency	Percentage	% Cumulative
Males	12	42.9%	42.9%
Females	16	57.1%	100.0%

Total	28	100.0%	100.0%

Pre-school aged, school aged and adolescents make up 50.0% (14/28) of the cases analyzed (Table 3). In the Brazilian population there is still a large number of children; however the population has aged in recent years, and this changed the age range distribution and the pyramid”s shape. In the demographic census of 2000, adults prevailed and it was also seen in the group we studied (42.9%).

**Table 3:** Individuals broken down by age.

Age range	Frequency	Percentage	% Cumulative
Infant	2	7.1%	7.1%
Pre-school	4	14.3%	21.4%
School	4	14.3%	35.7%
Adolescent	6	21.4%	57.1%
Adult	12	42.9%	100.0%

Total	28	100.0%	100.0%

The creation of inheritance patterns and discussing them with the genetic team has contributed to the analysis of the families (Figure 1, Figure 2, Figure 3, Figure 4, Figure 5, Figure 6, Figure 7, Figure 8, Figure 9). We observed intra and interfamily variability. The inheritance pattern analysis allowed us to suppose the occurrence of a new mutation in family 7 (II-2).

Proving that hearing disorder helps diagnose AS in the patients who had not undergone renal biopsy, making up a strict diagnostic criteria. Renal biopsy may be dubious in children and in women or, still, risky in cases of severe renal involvement.

In this sample we found 46.4% of DLX and 53.6% DA (Table 4). The intense participation of individuals (9/28) from family 1 may have contributed to this result. According to the literature, the most commonly pattern found was DLX and the analysis of inheritance patterns suggest an autosomal dominant type of AS.1 In approximately 85% of the inheritance patterns the disease is X-linked and the mutations were identified in the COL4A5 gene.2 The classification of the six AS subtypes^9^ is made up by the DA and DLX inheritance patterns. Patients with autosomal dominant As have large clinical variability and an enhanced molecular diagnosis can stress that this type is not so rare.^3^

**Table 4:** Individuals broken down by inheritance pattern.

Inheritance pattern	Frequency	Percentage	% Cumulative
AD	15	53.6%	53.6%
DLX	13	46.4%	100.0%

Total	28	100.0%	100.0%

We analyzed family members without clinical involvement and compared them to those who had renal alteration (Table 6). All of those who presented hearing alteration also had renal involvement. Among those with normal audiometric tests, 59.1% had renal involvement. There was an association between renal involvement and hearing disorder (p= 0.009) among the patients studied. 48% (12/25) of the patients in any stage of the renal disease had mild to moderate hearing disorder. 52% (13/25) of the patients at any stage of the renal disease did not have alterations in their audiometric tests.

**Table 6:** Individual breakdown as to renal involvement and hearing alteration.

	Renal Involvement		
Hearing alteration	Without alteration	With alteration	TOTAL
No alteration	9	13	22
Line %	40,9	59,1	100,0
Column %	100,0	52,0	64,7
With alteration (Mild and Moderate)	0	12	12
Line %	0,0	100,0	100,0
Column %	0,0	48,0	35,3
TOTAL	9	25	34
Line %	26,5	73,5	100,0
Column %	100,0	100,0	100,0

Exact Fisher Test  0,0094834717

Hearing alteration happens before the stage of renal failure, showing that it is not determined by uremia. It happens because of alterations in the basal membranes in the organs potentially affected by AS, according to the literature.^10^, ^11^, ^12^

HL is never seen without kidney symptoms.^12^

Hearing disorder progression suggests poor renal diagnosis, thus stressing the close tie between hearing alterations and renal failure in men and women.^13^, ^14^

Among the patients assessed in this sample, twelve had hearing disorders in their audiometric test: 11.5% (3/26) had mild disorder and 34.6% (9/26) had moderate hearing disorder (Table 5). As far as the age range of patients with mild hearing disorder (3/26) is concerned: one was of school age (11 years) and two were adults. Among the patients with moderate disorder (9/26) the age distribution is the following: one of school age (eight years), two teenagers (14 and 17 years) and six adults. This moderate involvement may happen because of the higher frequency of adults in this subgroup. HL happened in 46.1% (12/26) of the individuals evaluated. It is a frequent extra-renal finding in patients with AS.^11^ Hearing loss has been reported as one of the first symptoms of AS^6^, ^14^, and it may happen at ten years of age and it can be socially meaningless until the person reaches the second decade of life.^12^ The age of hearing loss can be associated with the type of mutation.^2^, ^15^, ^16^, ^17^

**Table 5:** Individuals broken down in relation to their level of hearing loss. (Classification from PARVING & NEWTON, 1995).

Hearing loss level	Frequency	Percentage	% Cumulative
Normal	14	53.9%	53.9%
Mild (21 to 40 dB HL)	3	11.5%	65.4%
Moderate (41 to 60 dB HL)	9	34.6%	100.0%

Total	26	100.0%	100.0%

There was no statistically significant correlation between the hearing disorder and the age of the patients studied (Table 9). Notwithstanding, in the general population we do not see hearing disorders in school-aged children, in adolescents and in adults, as noted in our findings. The chi^2^ test is vulnerable and the sample size added to the numerous possibilities analyzed can explain the fact that there was no association between the hearing disorder and age.

**Table 9:** Age range distribution as far as audiometric alterations are concerned.

Hearing loss severity				
Age ranges	Normal	Mild	Moderate	TOTAL
Pre-school	4	0	0	4
Line %	100,0	0,0	0,0	100,0
Column %	28,6	0,0	0,0	15,4
School	2	1	1	4
Line %	50,0	25,0	25,0	100,0
Column %	14,3	33,3	11,1	15,4
Adolescent	4	0	2	6
Line %	66,7	0,0	33,3	100,0
Column %	28,6	0,0	22,2	23,1
Adult	4	2	6	12
Line %	33,3	16,7	50,0	100,0
Column %	28,6	66,7	66,7	46,2
TOTAL	14	3	9	26
Line %	53,8	11,5	34,6	100,0
Column %	100,0	100,0	100,0	100,0

Chi-squared  g.l.  Likelihood

7,0847      6    0,3131

Still in this study group, 50.0% (13/26) of the patients had normal audiometric curves and 30.8% (8/26) had a mild descending curve in the high frequencies (Table 7). 11.5% (3/26) of the patients had a flat curve. The less frequent curve types were: sudden drop in high frequencies (3.8%) and U type (3.8%).

**Table 7:** Breakdown of the individuals as to the audiometric curve configuration.

Curve configuration	Frequency	Percentage	%Cumulative
Normal	13	50.0%	50.0%
Flat	3	11.5%	61.5%
Sudden drop in high frequencies	1	3.8%	65.4%
Mild drop in high frequencies	8	30.8%	96.2%
U Type	1	3.8%	100.0%

Total	26	100.0%	100.0%

Three audiometric curve types were seen in the 51 ears with hearing loss: cup shape (47.1%), descending (41.2%) and flat (11.7%).^18^

In the group assessed, we noticed 30.8% (8/26) of symmetrical losses and 19.2% had asymmetrical hearing loss (5/26) when comparing the two ears (Table 8). The literature reports that the hearing losses are symmetrical.^6^, ^10^, ^11^ This sample is small and does not allow us to establish a comparison of both ears.

**Table 8:** Breakdown of the individuals in relation to hearing loss symmetry.

Comparing the ears	Frequency	Percentage	% Cumulative
Normal	13	50.0%	50.0%
Symmetric	8	30.8%	80.8%
Asymmetric	5	19.2%	100.0%

Total	26	100.0%	100.0%

In this study we noticed 60.0% (6/10) of men with hearing alterations and 37.5% (6/16) of women. 62.5% (10/16) of women without hearing loss and 40.0% (4/10) of men in the same situation (Table 10). Literature reports that hearing loss happens more frequently in men than in women^15^; however, we did not observe any correlation between the hearing disorder and gender in this group studied.

**Table 10:** Hearing loss severity distribution as far as gender is concerned.

Hearing loss severity				
GENDER	Normal	Mild	Moderate	TOTAL
Males	4	1	5	10
Line %	40,0	10,0	50,0	100,0
Column %	28,6	33,3	55,6	38,5
Females	10	2	4	16
Line %	62,5	12,5	25,0	100,0
Column %	71,4	66,7	44,4	61,5
TOTAL	14	3	9	26
Line %	53,8	11,5	34,6	100,0
Column %	100,0	100,0	100,0	100,0

Chi-squared  g.l.  Likelihood

1,7230      2     0,4225

Prognosis has changed with the development of renal transplant, increasing the longevity of patients with AS and renal failure. Making HL development investigation and its rehabilitation relevant for the life quality of these individuals. There is a broad research field to be developed, with genetically modified animals or animal models, in order to understand the disease mechanisms involved in the hearing disorder and normal hearing. These same models can be employed in the study of new treatment modalities.

Not only the pathophysiological mechanisms of SNHL in AS still require additional studies, but also the clinical symptoms, hearing alterations and genotype-phenotype relation. The work of multidisciplinary groups from multiple centers shall bring about more information about the disease and its clinical course, diagnostic methods and treatment modalities. HL in AS is still a very exciting area for research by geneticists and otolaryngologists.

## CONCLUSIONS

- The inheritance patterns observed were AD and DLX. The distribution of inheritance patterns, in the present investigation, does not coincide with what is described in the literature.
- Hearing loss is frequently found in AS.
- All of those with hearing alterations also had renal involvement. There is a clear association between renal involvement and hearing alteration (p= 0.009).
- Audiometric curve types more commonly found in this sample were: mild drop in high frequencies and flat curves. The less frequently found curve types were: sudden drop and U-shaped type.
- There was no association between hearing alteration and age, found in the present study.
- We did not find correlations between these hearing alteration and gender in this study.
